# Alteration of glucose metabolism and expression of glucose transporters in ovarian cancer

**DOI:** 10.37349/etat.2024.00224

**Published:** 2024-04-24

**Authors:** Fatima Ben Ali, Zineb Qmichou, Mohamed Oukabli, Nadia Dakka, Youssef Bakri, Mohammed Eddouks, Rabii Ameziane El Hassani

**Affiliations:** Taipei Medical University, Taiwan, China; ^1^Laboratory of Biology of Human Pathologies (BioPatH), Faculty of Sciences, Mohammed V University in Rabat, Rabat 10001, Morocco; ^2^Medical Biotechnology Center, Moroccan Foundation for Advanced Science, Innovation and Research (MAScIR), Rabat 10001, Morocco; ^3^Department of Anatomical Pathology, Military Hospital of Instruction Mohammed V (HMIMV-R), Faculty of Medicine and Pharmacy, Mohammed V University in Rabat, Rabat 10001, Morocco; ^4^Team of Ethnopharmacology and Pharmacognosy, Faculty of Sciences and Techniques Errachidia, Moulay Ismail University of Meknes, Errachidia BP 509, Morocco

**Keywords:** Ovarian cancer, glucose transporters, aerobic glycolysis, diagnosis biomarker, prognosis biomarker, chemotherapy resistance

## Abstract

Aerobic glycolysis also known as the Warburg effect, remains a hallmark of various cancers, including ovarian cancer. Cancer cells undergo metabolic changes to sustain their tumorigenic properties and adapt to environmental conditions, such as hypoxia and nutrient starvation. Altered metabolic pathways not only facilitate ovarian cancer cells’ survival and proliferation but also endow them to metastasize, develop resistance to chemotherapy, maintain cancer stem cell phenotype, and escape anti-tumor immune responses. Glucose transporters (GLUTs), which play a pivotal role as the rate-limiting step in glycolysis, are frequently overexpressed in a variety of tumors, including ovarian cancer. Multiple oncoproteins can regulate GLUT proteins, promoting tumor proliferation, migration, and metastasis, either dependent or independent of glycolysis. This review examines the alteration of GLUT proteins, particularly GLUT1, in ovarian cancer and its impact on cancer initiation, progression, and resistance to treatment. Additionally, it highlights the role of these proteins as biomarkers for diagnosis and prognosis in ovarian cancer, and delves into novel therapeutic strategies currently under development that target GLUT isoforms.

## Introduction

Ovarian cancer (OC) is one of the most frequent and lethal gynecological cancers, with a 5-year survival rate below 45% [1]. It comprises heterogeneous groups of diseases that vary in origin, pathogenesis, and prognosis. Epithelial ovarian carcinomas (EOC) constitute the most frequent type of OC (90% of OC) [2]. Based on the updated 2020 World Health Organization (WHO) classification, a minimum of five primary types of ovarian carcinomas have been distinguished according to histopathological features, immuno profiling, and molecular analysis, such as high-grade serous ovarian carcinoma (HGSOC), endometroid carcinoma (EC), clear cell carcinoma (CCC), low-grade serous ovarian carcinoma (LGSOC), and mucinous carcinoma (MC) [3]. The most common subtype of EOC is HGSOC, accounting for over 70% of cases [4]. HGSOCs are characterized by a late-stage diagnosis, result in a poor prognosis, and are associated with high levels of genomic instability, primarily attributed to the frequent tumor protein 53 (*TP53*) mutations (96% of cases) and inherited mutations in breast cancer (BRCA) susceptibility genes 1 (*BRCA1*) and *BRCA2* [5].

**Figure 1:**
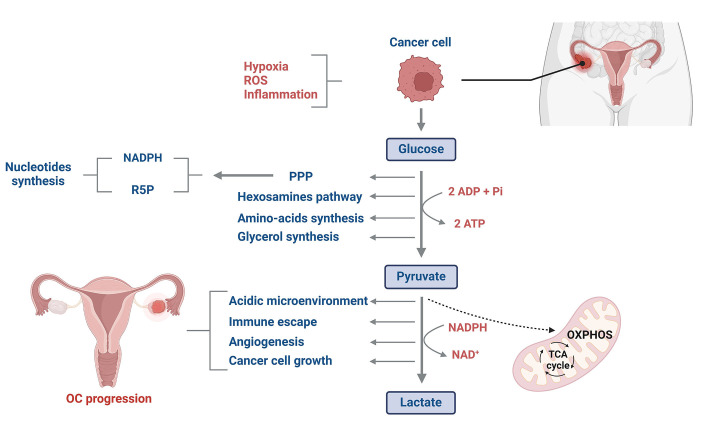
Glucose metabolism reprogramming in OC cells. Glucose metabolism in OC cells differs significantly from that of normal cells. Despite tumor cells having higher energy demands compared to normal cells, they predominantly generate energy through glycolysis rather than OXPHOS in the mitochondria. This phenomenon, often referred to as aerobic glycolysis or the Warburg effect, allows cancer cells to produce ATP more rapidly as well as to synthesize glycolytic intermediates for anabolic processes. Additionally, the elevated production of lactate significantly increases the acidity of the tumor microenvironment, creating a favorable environment for cancer cell proliferation while potentially inhibiting the immune cell system. ROS: reactive oxygen species; TCA: tricarboxylic acid cycle; ADP: adenosine diphosphate; Pi: inorganic phosphate; NAD: nicotinamide adenine dinucleotide. This figure was created with BioRender.com

Alongside genetic and molecular signatures, notable metabolic changes are associated with ovarian malignancies. The dysregulation of glucose metabolism in cancer cells was described for the first time by Warburg [6] in the 1920s. Tumor cells consume more energy compared to normal cells. In fact, they produce energy through glycolysis rather than oxidative phosphorylation (OXPHOS) in the mitochondria, even under normoxic conditions [7]. Aerobic glycolysis is probably by cancer cells because it confers a quicker ATP production (10–100 times faster than OXPHOS) [8, 9]. Thus, a higher rate of ATP could be generated in the tumor microenvironment, where energy resources are constrained (Figure 1) [10]. Besides, glucose is primarily consumed by cancer cells to provide glycolytic intermediates for anabolic processes [11]. Glucose-6-phosphate can undergo oxidation by glucose-6-phosphate dehydrogenase (G6PD), resulting in the production of reduced nicotinamide adenine dinucleotide phosphate (NADPH) and ribose-5-phosphate (R5P) through the pentose phosphate pathway (PPP) [12]. NADPH and R5P are essential for nucleotide synthesis and play critical roles in sustaining biosynthetic reactions and preserving the cell’s redox capacity (Figure 1) [13]. In addition, the production of high levels of lactate decreases mitochondrial activity and increases the acidity of the tumor microenvironment, enabling cancer cells to evade immune defenses and support their uncontrolled proliferation to drive cancer progression (Figure 1) [14].

Factors such as hypoxia, oxidative stress, and inflammation are known to redirect cell metabolism toward aerobic glycolysis and enhance glucose caption [15]. Indeed, an increased rate of glucose uptake resulting from metabolic process alterations has been identified in ovarian CCC. This finding was primarily attributed to the overexpression of various molecular signatures, particularly the hepatocyte nuclear factor-1B (*HNF1B*) and facilitative glucose transporters (GLUTs, GLUT1) protein [16]. The results discussed in this review are from several studies carried out using representative models of OC as well as: human OC cell lines (SKOV3, A2780, HO8910, 3AO); the human ovarian epithelial cell line HOSEpiC; and human ovarian carcinomas and matched normal ovarian tissue samples. Warburg effect has further been shown to play a role in the progression of OC. Moreover, the robust activation of phosphoinositide 3-kinase (PI3K)/protein kinase B (Akt)/mechanistic target of rapamycin (mTOR) signaling pathways has been described to promote epithelial ovarian carcinogenesis through the upregulation of GLUT proteins expression resulting in elevated glucose influx [17]. Besides, glucose metabolism alteration could display a significant role in the invasiveness and metastasis of epithelial and granulosa OC cells [18]. In this review, recent findings concerning GLUTs and disruption of glucose metabolism in OC are discussed as well as diagnostic and therapeutic approaches.

## Role of facilitative GLUTs and their expression in OC

### Role of facilitative GLUTs

Glucose overwhelmingly serves as the primary source of ATP generation for most mammalian cells. Through a series of biochemical processes, a single glucose molecule has the potential to yield around 38 ATP molecules that cells can utilize [19]. Glucose molecules are polar and cannot traverse the lipid bilayer of the cell membrane on their own. Therefore, facilitative transporters are necessary to enable their entry into cells [20]. Facilitative GLUTs are a family of proteins that catalyze glucose uptake and entry into nearly all mammalian cells across an electrochemical gradient. They are encoded by a superfamily known as solute carriers 2 genes (*SLC2*) with 14 GLUT proteins (GLUT1–14) corresponding to 14 *SLC2* genes (*SLC2A1–14*) [21, 22]. *SLC2* genes encode for different protein families that mediate the transport of nutrients, ions, or other metabolites across cell or organelle membranes [23].

Based on their sequence homology and substrate selectivity, GLUT protein members have been categorized into three classes [24]. Class 1 comprises GLUT1–4 and GLUT14; class 2 comprises GLUT5, GLUT7, GLUT9, and GLUT11; and class 3 comprises GLUT6, GLUT8, GLUT10, GLUT12, and GLUT13/H^+^-myo-inositol co-transporter (HMIT) [25]. The sizes of GLUT proteins vary from 492 to 524 amino acids. The amino acid sequences show 39–65% identity between the well-characterized human GLUT1–5 and 28% identity between GLUT1 and all other GLUTs [26]. Mammalian GLUT members exhibit varying affinity for glucose as well as other sugars like fructose or mannose [26]. Besides, they are characterized by their tissue-specific expression and function (Table 1). Typically, the level of expression correlates with the rate of cellular glucose metabolism [27].

**Table 1:** Expression of main GLUT isoforms related to OC

GLUT isoforms	Major site of expression	Nature of transport	References
GLUT1	Placenta, brain, muscles, kidney, and colon	Basal continuous uptake	[26]
GLUT2	Liver, B-cell, kidney, and small intestine	Two-way transportation of glucose	[25]
GLUT3	Many tissues, including brain, placenta, and kidney	Neuronal uptake	[28]
GLUT4	Skeletal muscle, heart, and brown and white fat	Insulin-responsive	[29]

The GLUT1 and GLUT3 isoforms are most likely accountable for the continuous basal glucose uptake. They are present in almost all tissues and are particularly highly expressed in the brain and erythrocytes [28]. GLUT2 is a low affinity and high Km isoform that mediates the two-way transportation of glucose into hepatocytes and from absorptive epithelial cells into the bloodstream in the small intestine and kidney [25]. GLUT5 is a fructose transporter [30]. GLUT4 functions as an “insulin-responsive” isoform and is expressed in skeletal muscle, heart, and white and brown adipose tissue as well [29]. Certain tissues can express multiple GLUTs, as seen in muscles where the presence of GLUT3, GLUT4, GLUT5, GLUT10, and GLUT11 has been documented [31–33].

### GLUTs expression in cancer

Alteration of GLUT expression has been associated with many cancer types [34]. Indeed, cancer cells often alter the expression of GLUTs to enhance glucose influx, thereby ensuring their anarchic proliferation. Overexpression of GLUT1 and/or GLUT3 is linked to poor survival outcomes across a variety of investigated cancer types, including breast carcinoma, lung adenocarcinoma, colorectal carcinoma, ovarian carcinoma, squamous cell carcinoma, and glioblastoma [15, 22, 35, 36]. Moreover, GLUT1 overexpression is associated with poor prognosis following surgical resection in colorectal tumors [37] as well as in cases of esophageal and oral squamous carcinoma [38, 39].

**Figure 2:**
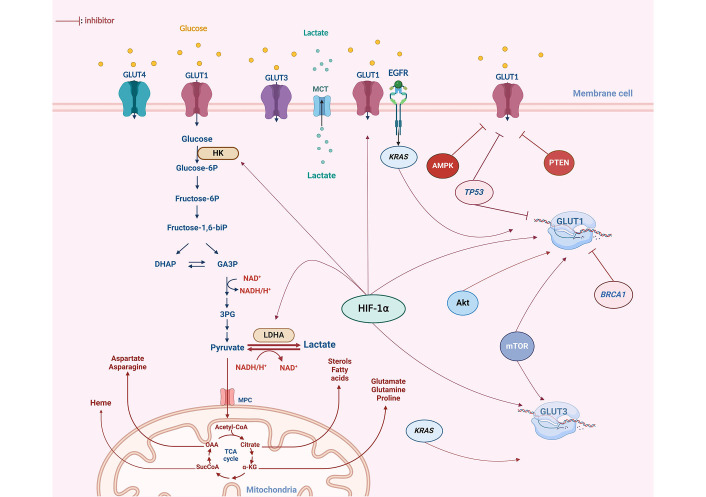
Glucose metabolism alteration and dysregulation of GLUTs expression in OC. The GLUT1 isoform exhibits notably high expression in OC. Various oncogenes have been identified as contributors to the upregulation of GLUT isoforms in OC, particularly GLUT1. The HIF-1α plays a crucial role in modulating GLUT1 expression, acting as a key factor in the upregulation of both transcription and the translocation of GLUT1 to the plasma membrane. Mutations in oncogenes such as *KRAS*, *Akt*, and *mTOR* have also been reported to enhance glycolysis by increasing GLUT1 expression in response to the elevated energy demands of cancer cells. Furthermore, other key glycolytic enzymes, including hexokinase (HK) and lactate dehydrogenase (LDHA), have been observed to be upregulated in OC. Tumor suppressors such as *TP53* and *BRCA1* inhibit the expression of GLUT proteins at both a transcriptional and cytoplasmic level. However, these two genes are found to be mutated in 96% and 20% of OC cases, respectively. DHAP: dihydroxyacetone phosphate; GA3P: glyceraldehyde 3 phosphate; 3PG: 3 phospo-glycerate; MCT: monocarboxylate transporter; EGFR: epidermal growth factor receptor; AMPK: adenosine monophosphate-activated protein kinase; PTEN: phosphatase and tensin homolog; glucose-6P: glucose-6 phosphate; biP: biphosphate; MPC: mitochondrial pyruvate carrier; CoA: coenzyme A; OAA: oxaloacetate; SucCoA: succinyl-coenzyme A; α-KG: alpha-ketoglutarate. This figure was created with BioRender.com

Modulation of GLUT expression is triggered by a range of molecular pathways (Figure 2). Hence, altered oncogene activation, loss or lack of tumor suppressor gene functions, and mitogen stimulation are all reported to increase the expression of GLUT proteins and enhance the rate of glucose uptake in cancer tissues [36]. Serine/threonine kinase Akt protein, known as a mediator of GLUT4 translocation to the plasma membrane, has been demonstrated to induce overexpression of GLUT1 and GLUT3 in cancer cells [40], therefore, promoting glucose transport stimulation. Additionally, in myeloma cells, the presence of activated Akt substrate of 160 kDa (AS160), an effector downstream of Akt, has been linked to the translocation of GLUT4 to the plasma membrane, contributing consequently to the Warburg effect by intensifying glucose availability [41]. Likewise, the hypoxia-inducible factor (HIF) 1α (HIF-1α) has been reported to modulate GLUT1 and GLUT3 transcription [34]. Besides, an elevated expression of HIF-1α and GLUT3 was found to be correlated with chemotherapy resistance in acute myeloid leukemia [42]. Mutations in Kirsten rat sarcoma virus (*KRAS*) and V-Raf murine sarcoma viral oncogene homolog B (*BRAF*) oncogenes are also implicated in the alteration of GLUT expression [43]. Indeed, colorectal cells harboring these mutations display an increased glucose uptake resulting from GLUT1 upregulation, aerobic glycolysis, and a heightened survival rate even under low-glucose conditions [43].

Downregulation of GLUT protein isoforms is mediated by a variety of anti-proliferative genes essentially the tumor suppressor gene *TP53* [44]. The activation of *TP53* following a genotoxic or metabolic stress signal resulted in a subsequent modification of *TP53*-dependent gene expression, including inhibition of GLUT protein expression [45, 46]. However, *TP53* was found to be mutated in most of the malignant tumors, thus these actions cannot be carried out [47].

### GLUTs expression in OC

Aberrant expression of GLUT1 has been documented in specimens of epithelial OC [48, 49]. This expression pattern exhibited variations across distinct histological types [50]. Specifically, in serous ovarian carcinomas (SOC), the level of GLUT1 expression was markedly higher compared to ovarian CCC [51]. However, these differences in GLUT1 expression were not significant between SOC and mucinous ovarian carcinomas (MOC), nor between HGSOC and LGSOC [49]. Furthermore, the expression of GLUT1 protein was found to correlate with the clinical stage of the disease [50]. This is evidenced by a gradual increase in GLUT1 expression observed from borderline to malignant tumors as well as from well-differentiated to poorly differentiated ovarian carcinoma [48]. It is noteworthy that neither benign lesions nor normal ovarian epithelial cells exhibited specific GLUT1 protein expression [49, 52]. These findings suggest the involvement of GLUT1 transporter in the malignant transformation and metastasis of ovarian tumors. This involvement is likely attributed to its capacity to facilitate glucose uptake of tumor cell clones with an increased energy requirement [53]. Additionally, cancer cells exhibited strong immunohistochemical staining of the plasma membrane, while the stromal component showed no staining. Besides, the intensity of the reaction varied among individual cells and was more pronounced in regions distal from blood vessels [52].

On the contrary, GLUT3 immunohistochemistry revealed homogenous faint staining in both benign and malignant ovarian tissues [49]. Furthermore, GLUT3 expression remained consistent across various histological types, and there were no observed correlations between GLUT3 expression and the clinical stage of the disease [50].

Only a limited number of studies have explored the expression pattern of GLUT4 in cancer cells. GLUT4 expression levels were observed to be mild to moderate across various ovarian subsets [50]. Nevertheless, it is worth noting that mucinous and clear cell adenocarcinoma exhibited a higher rate of GLUT4 expression when compared to other histological types [54].

The previously cited reports highlight a significant alteration in GLUT1 expression compared to other GLUTs (Figure 2). Despite this observation, the underlying mechanisms of GLUT1 induction remain poorly understood [55]. However, it is well-established that the HIF plays a crucial role in modulating GLUT1 expression and may serve as a key factor in the upregulation of GLUT1 transcription [56]. OC is particularly prone to metastasizing to the peritoneum through tumor survival and proliferation in peritoneal fluid. Thus, cancer cells can experience oxygen deprivation within the tumor microenvironment [57]. Consequently, hypoxia exerts a notable influence on adaptive mechanisms within OC [49]. Indeed, these adaptive responses to hypoxia may provide a plausible explanation for the elevated expression of GLUT1 in malignant ovarian tumors, particularly in areas distant from the stroma [53]. This phenomenon could potentially account for the heterogeneous expression of GLUT1 within OC tissue [58]. Moreover, genetic alterations, such as *TP53* mutations recognized as triggers in the development of OC, especially the high-grade serous subtype, and are also known to contribute to the upregulation of GLUT1 [59]. This could potentially explain the observed positive correlation between GLUT1 overexpression and the clinical stage of OC.

## GLUTs as diagnosis and prognosis biomarkers of OC

Due to the non-specific nature of OC symptoms, and the lack of effective early-stage screening, the diagnosis remains a significant challenge in the management of the disease [60]. It is worth noting that early diagnosis of OC can increase the 5-year survival rate to as high as 90% compared to only 30% when cancer is diagnosed at an advanced stage [61]. The current diagnostic approach for ovarian lesions involves gynecological evaluation, transvaginal ultrasonography (TVUS), and the assessment of specific serum tumor markers, such as cancer antigen 125 (CA125) [62]. Nonetheless, despite the high sensitivity and specificity exhibited by this approach in detecting malignant ovarian neoplasms, its accuracy in achieving an exact differential diagnosis is limited, primarily due to the morphological similarities between benign and malignant ovarian lesions [63].

Currently, positron emission tomography (PET) scans have become a crucial tool for cancer screening [64]. Based on the introduction of a glucose analog called 18-fluorine-fluorodeoxyglucose (18F-FDG), PET imaging is used to provide insights into both the anatomical and metabolic aspects of tumors [65]. 18F-FDG is transported into glucose-consuming cells through GLUTs, particularly GLUT1, and is retained as 18F-FDG 6-phosphate following phosphorylation by HK, typically type II [66].

The uptake of 18F-FDG has been linked to parameters such as tumor proliferation rate, tumor grade, and the expression of GLUTs, all of which serve as critical biomarkers influencing response to chemotherapy, prognosis, and overall survival rates in cases of malignant diseases [67]. Indeed, the introduction of 18F-FDG PET/computed tomography (CT) has marked a significant advancement in diagnostic accuracy and has had a substantial impact on patient management [68].

18F-FDG PET/CT demonstrates relatively high diagnostic accuracy when distinguishing between malignant and benign ovarian tumors [69]. Furthermore, there is compelling evidence indicating a positive correlation between 18F-FDG uptake and GLUT1 expression in OC [70]. Indeed, studies have consistently reported a gradual increase in 18F-FDG uptake, as quantified by the standardized uptake value (SUV), from well-differentiated to poorly differentiated OCs, and from borderline to malignant tumors, closely aligning with GLUT1 expression intensity [57, 71]. This observation implies that the potential relationship between GLUT1 expression and 18F-FDG uptake is linked to cellular growth during carcinogenesis and the malignant transformation of epithelial ovarian tumors [52]. Consequently, they could hold significant value across various stages of cancer management, including the diagnosis and staging of ovarian lesions [71]. Besides, increased GLUT1 expression has been associated with poor survival outcomes in patients with OC [72]. Thus, GLUT1 status could serve as an independent prognostic factor, particularly influencing the response to chemotherapy in advanced ovarian carcinoma [73].

In summary, GLUT expression in OC serves as an indicator not only of the metabolic requirements and vascular support within tumors but also carries significant clinical implications for patient diagnosis and prognosis. Hence it plays a pivotal role in assessing tumor response to therapeutic interventions and evaluation of survival outcomes of OC patients.

## Targeting GLUT overexpression and metabolic reprogramming for new therapeutic approaches against OC

Despite the emergence of new therapeutic strategies against OC, including poly-ADP ribose inhibitors (PARPi) and folate receptor α inhibitors, significant advancements in OC prognosis remained elusive, primarily because of the widespread chemoresistance observed in the majority of cases of epithelial OC [74]. Drug resistance remains a complex issue that involves several molecules and pathways. Therefore, additional efforts are currently required to comprehend and overcome molecular and genetic changes responsible for drug resistance.

The metabolic phenotype of OC cells depends on their ability to adjust and change their metabolism in response to different situations [75]. This plasticity enables tumor cells to reprogram their metabolism under normoxic and hypoxic, quiescent and proliferative conditions [76]. In fact, researchers suggest that tumors acquire chemotherapy resistance by displaying high plasticity to metabolic reprogramming, specifically, aerobic glycolysis (Warburg effect) [11]. This adaptive metabolic plasticity contributes to the development of unique phenotypic traits in tumor cells [77].

GLUT proteins constitute the first rate-limiting step of glycolysis and are signiﬁcantly upregulated in EOC particularly HGSOC histotype [78]. Extensive research has investigated the role of GLUTs inhibition in OC subsets [79]. Although the Food and Drug Administration (FDA) has not yet approved any glycolysis inhibitors for OC treatment, several inhibitors have shown promise in preclinical and clinical studies, and further research is needed to determine their safety and efficacy in OC remission (Table 2) [80]. The glucose analog 2-deoxy-*d*-glucose (2-DG) has been explored not only for cancer diagnosis but, also as a potential cancer inhibitor [81]. 2-DG undergoes phosphorylation by HK to form 2-DG-6-phosphate (2DG6P) and accumulates within the cell [82]. This accumulation hinders the glycolytic process from utilizing its usual substrate, thereby reducing energy generation. Consequently, the decrease in cellular ATP levels disrupts cell cycle progression and ultimately results in cell death [83]. Interestingly, 2-DG has been reported to enhance the growth inhibitory effect of cisplatin in both SKOV3 and TOV21G (ovarian adenocarcinoma cell models) cell lines [84]. Besides, the combination of cisplatin and 2-DG resulted in robust activation of the intrinsic apoptotic pathways, such as caspase-9. Hence, a significantly increased proportion of apoptotic cells was observed in both cell lines [84]. Furthermore, 2-DG was reported to induce G1 phase cell cycle arrest and to reduce glycolytic activity in IGROV-1, SKOV3, and Hey cell lines [85]. However, due to its severe side effects, it was arrested in phase Ι clinical trials [77, 85].

**Table 2:** Overview of highly potent inhibitors of GLUT isoforms overexpressed in OC

**GLUT inhibitors**	**GLUT isoforms**	**Clinical phase**	**Main cell/animal models**	**Effective inhibitory concentration**	**Reference**
2-DG	GLUT1	Phase Ι clinical trials	SKOV-3/TOV21	20 mmol/L	[84, 85]
RV	GLUT1	*In vitro* assays	OVCAR3/OAW42	100 µmol/L	[86]
Ciglitazone	GLUT1	*In vitro* and *in vivo* assays	A2780, NSG mice	1 µmol/L	[87]
Silibinin	GLUT4	*In vitro* assays	SKOV-3	100–200 µmol/L	[88]
BAY-876	GLUT1	*In vitro* and *in vivo* assays	SKOV-3/OVCAR-3/A2780 patient-derived xenograft	60–180 nmol/L	[89]
STF31	GLUT1	*In vitro* assays	OVCAR5/TOV112D/OVCAR3	0.8–1.5 µmol/L	[51]

Several natural compounds have been the focus of numerous studies due to their potential anticancer properties and low toxicity. Among these compounds, nutraceutical resveratrol (RV), known for its anti-diabetic properties, has been investigated for its potential anticancer effects [85]. In the context of OC, RV has shown promise in regulating the glycolytic phenotype of HGSOC cells (OVCAR3 and OAW42) by retaining the GLUT1 protein in a perinuclear localization [86]. GLUT1 translocation block leads to glucose shortage which has been observed to enhance autophagy in OC cells [86]. Additionally, RV suppresses the pro-migratory effects of interleukin-1 (IL-1), which is abundant in the ascitic fluid of OC patients [90]. These findings hold the potential to enhance the response to platinum therapy, improve overall survival rates, and enhance clinical outcomes for OC patients. Besides, studies show the potential effectiveness of Silibinin (natural flavonolignan) in glycolysis modulation. Silibinin inhibits glucose uptake by a direct interaction with GLUT4 isoform [91]. Silibinin effectively inhibits cell proliferation in SKOV-3 cells, and its combination with paclitaxel demonstrates greater efficacy compared to paclitaxel treatment alone [88]. Notably, the expression of apoptosis-related genes, *TP53* and cyclin-dependent kinase inhibitor 1 *(CDKN1A)*, was significantly upregulated in cells treated with paclitaxel combined with silibinin when compared to the non-treated group [88].

Small synthesized or bioactive molecules were further reported to exhibit an inhibitory effect of the Warburg effect. Indeed, BAY-876 inhibitor [N4-1-(4-cyanobenzyl)-5-methyl-3-(trifluoromethyl)-1*H*-pyrazol-4-yl-7-fluo­roquinoline-2,4-dicarboxamide] demonstrates notable effectiveness in suppressing GLUT1 expression resulted in decreased glycolysis and a significant reduction of tumor growth in both *in vivo* and *in vitro* studies [89]. It is important to note that the effectiveness of GLUT1 inhibition varied between ovarian cell lines. In OVCAR3 and SKOV-3 cells, inhibition of cell growth was significant with a half maximal inhibitory concentration (IC_50_) value of up to 60 nmol/L to 188 nmol/L, while A2780 (adenocarcinoma cells model) were resistant to treatment. Besides, in SKOV-3 xenograft mice, daily administration of 4.5 mg/kg BAY-876 resulted in a 68% reduction in tumor volume after two weeks [87]. These findings suggest that the inhibition of BAY-876 is dependent on the cell line. Furthermore, ciglitazone, a peroxisome proliferator-activated receptor gamma (PPARγ) agonist, has demonstrated the ability to induce cell death in OC [92]. This effect is achieved through the inhibition of GLUT1 protein expression in A2780 OC cells, with effective inhibitory concentrations as low as 1 µmol/L to 10 µmol/L. Indeed, the inhibitory effect has been validated *in vivo* using OC cells xenografted into non-obese diabetic (NOD) scid gamma (NSG) mice [92]. Besides, ciglitazone has been shown to reduce the expression of β-catenin while increasing AMPK phosphorylation levels [93]. AMPK activation plays a role in suppressing cancer cell growth by inhibiting β-catenin signaling activity [94]. These findings suggest that ciglitazone induces cell death by simulating conditions of glucose deprivation and it can be employed as an adjuvant drug for serous OC treatment. Subsequently, the recently developed compound 4-4-(1,1-dimethylethyl) phenyl-sulfonyl-amino-methyl-*N*-3-pyridinylbenzamide (STF31), has attracted attention for its capacity to disrupt glycolytic metabolism through its ability to bind directly to the GLUT1 transporter [95]. Interestingly, when combined with metformin (an inhibitor of mitochondrial respiratory chain complex 1), STF31 exhibited enhanced cytotoxicity in both platinum-sensitive and platinum-resistant OC cells [51, 96]. This combination approach led to a significant antitumor effect, as evidenced by low synergistic confidence interval (CI) values [51]. These findings provide further support for the notion that dual inhibition of these two energy pathways holds promise as a therapeutic strategy for OC evasion.

Overall, targeting metabolism, especially in combination with chemotherapeutic drugs, is essential to improve therapies and may help overcome drug resistance in OC [72]. Metabolic reprogramming, which encompasses disrupted glycolysis, increased ATP generation, and cell-death escape mechanisms, stands as a principal contributor to therapeutic resistance in OC cells [97]. GLUT inhibitors have proved a significant efficacy in the regulation of glycolysis and cancer cell proliferation rates [98]. Indeed, targeting glycolysis-related genes in particular GLUT1 is a promising strategy to re-sensitize OC cells toward chemotherapy. However, the heightened similarity and the possibility of compensation among different isoforms within the GLUT family pose a substantial challenge in achieving significant inhibition of glycolysis rate within cancer cells [80]. Moreover, glycolysis is a process commonly found in normal cells. For instance, red blood cells derive their energy from glycolysis while delivering oxygen to other cells in the body [99]. Also, endothelial cells exhibit high expression of GLUT1 on their surface and derive around 85% of their ATP supply from glycolysis [100]. Consequently, inhibiting glycolysis could impair the capacity of red blood cells to transport oxygen and hinder blood vessel formation and endothelial cell angiogenesis causing eventually the death of normal cells [101]. Therefore, developing GLUT and glycolysis inhibitors that preserve normal cell function is of crucial importance.

## BRCA and OC

*BRCA1* and *BRCA2* are the most hallmarks in breast and OC [102]. Mutations in these genes highly predispose to hereditary forms of cancer for both cancer types. Indeed, *BRCA1* mutation carriers have up to 46% risk of developing epithelial OC compared to the general population where the lifetime risk is only 1.4% [102, 103]. *BRCA1* is a tumor suppressor gene known for its function in double-stranded DNA breaks (DSBs) reparation through homologous recombination [104]. Loss or lack of *BRCA1* functions promotes carcinogenesis induced by high genomic instability [105]. A limited number of studies have investigated the role of *BRCA1* in cancer metabolism. However, *BRCA1* has been found to play a non-negligible role in metabolism pathways regulation, specifically in the modulation of glycolytic phenotype. In fact, silencing of the *BRCA1* gene has been demonstrated to increase the rate of glycolysis in both cancerous and non-cancerous ovarian cells [106]. Likewise, *BRCA1* was reported to reverse the Warburg effect via the TCA cycle and OXPHOS activation in BRCA cells [107]. Furthermore, higher levels of GLUT1 expression were observed in cancer cells carrying germline mutations in the *BRCA1* gene [56]. Indeed, cancer cells with a non-mutant *BRCA1* gene were shown to suppress glycolysis by inhibiting the expression of the *GLUT1* gene [107]. Notably, patients with *BRCA* mutations have shown a favorable response and improved survival, particularly when treated with intraperitoneal (IP) chemotherapy.

## Conclusions

Cancer cell metabolism presents a new promoting field in cancer research. Metabolism reprograming and altered glycolysis pathways in OC have been well validated. Overexpression of GLUT proteins especially GLUT1 is found to be highly correlated with tumor malignancy and clinical stage of OC. This is may have a significant value in the diagnosis, prognosis, and treatment of OC. However, more investigations are still needed to better understand the key effectors of metabolism-induced ovarian carcinogenesis. Hence, the exploration of new genetic and molecular alterations that rely on ovarian tumorigenesis seems to be advantageous. Therefore, the BRCA mutation status in OC patients seems to be critical in the management of the disease. Nevertheless, further researches are needed to explore its relationship to the glycolytic phenotype and metabolic changes. Thus, investigating *BRCA1* profiles regarding metabolic changes in OC patients could reveal new approaches for OC management.

## Abbreviations

18F-FDG: 18-fluorine-fluorodeoxyglucose
2-DG: 2-deoxy-d-glucose
Akt: protein kinase B
AMPK: adenosine monophosphate-activated protein kinase
BRCA: breast cancer
BRCA1: breast cancer susceptibility genes 1
CCC: clear cell carcinoma
EOC: epithelial ovarian carcinomas
GLUTs: glucose transporters
HGSOC: high-grade serous ovarian carcinoma
HIF-1α: hypoxia-inducible factor 1α
HK: hexokinase
KRAS: Kirsten rat sarcoma virus
mTOR: mechanistic target of rapamycin
NADPH: nicotinamide adenine dinucleotide phosphate
OC: ovarian cancer
OXPHOS: oxidative phosphorylation
PET: positron emission tomography
R5P: ribose-5-phosphate
RV: resveratrol
SLC2: solute carriers 2 genes
STF31: 4-4-(1,1-dimethylethyl) phenyl-sulfonyl-amino-methyl-*N*-3-pyridinylbenzamide
TP53: tumor protein 53

## Declarations

### Copyright

© The Author(s) 2024.
